# Detecting Chemical Vapor Diffusion through Firefighter Turnout Gear

**DOI:** 10.3390/ijerph18094833

**Published:** 2021-04-30

**Authors:** Michelle A. Corbally, Mary R. Williams, Jessica N. Chappell, Michael E. Sigman

**Affiliations:** 1 Department of Chemistry, University of Central Florida, P.O. Box 162367, Orlando, FL 32816-2366, USA; macorbally@Knights.ucf.edu; 2 National Center for Forensic Science, University of Central Florida, P.O. Box 162367, Orlando, FL 32816-2367, USA; Mary.Williams@ucf.edu (M.R.W.); j_chappell@knights.ucf.edu (J.N.C.)

**Keywords:** firefighters, carcinogens, fire debris analysis, gas chromatography–mass spectrometry, target factor analysis

## Abstract

Firefighters are exposed to burning materials that may release toxic partial combustion and pyrolysis products into the environment, including compounds listed as priority pollutants by the United States Environmental Protection Agency (EPA). A novel passive sampling dosimeter device containing firefighter turnout gear as a diffusion membrane and an activated charcoal strip (ACS) for volatile analyte collection was designed and used to monitor potential exposures of firefighters to volatile organic compounds. Solvent extracts from the ACS and turnout gear diffusion layer were analyzed using Gas Chromatography–Mass Spectrometry (GC-MS) to determine the diffusion of compounds from burned substrates through firefighter turnout gear and compound adsorption to the turnout gear. The compounds in these samples were identified using target factor analysis (TFA). An activated carbon layer (ACL) was added to the dosimeter between the turnout gear and the ACS. The presence of combustion and pyrolysis compounds identified on the ACS in the dosimeter was reduced.

## 1. Introduction

Firefighters are consistently exposed to atmospheres in structure fires containing various combinations of partial combustion and pyrolysis components from substrates and occasionally ignitable liquids. According to the National Fire Protection Association (NFPA) 1971–18 performance specifications, firefighter turnout gear is structurally developed to protect firefighters from physical threats in a fire, but volatile carcinogens may diffuse through the material [1]. Current procedures involving turnout gear and chemical vapors describe the necessity for extra precautions to take place during a chemical, biological, radiological, or nuclear (CBRN) hazardous materials situation, but do not outline potential deterrents to protect from carcinogens present in vapors during a normal fire [2].

Carcinogens are defined by the United States Environmental Protection Agency (EPA) as a compound that can cause cancer in a human, or animal [3]. The EPA has compiled a list of priority pollutants found in the environment that are considered carcinogenic to humans and animals [4]. Previous studies have shown firefighters are often exposed to many of the pollutants on the priority pollutant list including, but not limited to, metals, polyaromatic hydrocarbons, and halogenated vapors that can have acute effects on the human body [5,6]. A meta-analysis of 32 studies was performed in 2006 to provide a quantitative summary of risk estimates for various cancers diagnosed in firefighters [7]. The study demonstrated that firefighters have a higher risk of developing multiple myeloma, non-Hodgkin lymphoma, prostate, and testicular cancers than the general public [7].

Structural and vehicular fires are known to release chemical vapors and particles that are carcinogenic in nature to humans [5,6]. The Ignitable Liquid Reference Collection (ILRC) and Substrate databases developed by the National Center for Forensic Science (NCFS) and the Technical/Scientific Working Group for Fire and Explosions (T/SWGFEX) contain data of ignitable liquids and burned substrate reference materials. Five major compounds, typically the most abundant compounds are identified for each reference material. To identify these major compounds, a mass spectral library that currently contains 295 compounds was produced (NCFS library). Mass spectral comparison with the library was performed using ChemStation software with identification performed by an analyst applying mass spectral match and retention time shift criteria. The frequency at which one of the 295 compounds in the library was identified has previously been reported by Akmeemana et al. [8]. In the study reported here, a novel application for identification of compounds utilizing target factor analysis (TFA) in an automated process was used with the NCFS library to attempt identification of all library compounds in the reference materials. A portion of the identified compounds are contained in the EPA priority pollutant list [9,10].

Fent and Evans previously examined firefighter exposure to chemical vapors during suppression of vehicle fires by collecting vapor samples using a summa cannister [11]. The work focused specifically on the use of self-contained breathing apparatus by firefighters. Fent and coworkers later examined the exposure of firefighters to particulates and chemical vapors, including polycyclic aromatic hydrocarbons and benzene, during the extinguishment of structure fires [12]. Particulate samples were collected using virtual impactor sampling devices and personal vapor samples were collected using mechanical pumps drawing air samples through tubes containing XAD resin. In many cases the air sampling pumps failed at some point during the experiments. A previous study performed by Barker et al. used a passive sampling dosimeter to test the performance of a selective permeable membrane in preventing chemical vapors from diffusing through turnout gear [13]. Methyl salicylate, the chemical vapor simulant, was used to test the selective permeable membrane developed [13]. In the study by Barker, commercially available dosimeters were placed against the skin of test subjects in several locations.

In this work, we utilize passive air sampling dosimeters to monitor chemical vapors having passed through a sampling ‘membrane’ composed of three layers of firefighter turnout gear. Unlike summa canisters, passive sampling devices rely on concentration gradients across a membrane, rather than actively sucking air into a previously evacuated container. Passive sampling devices do not rely on mechanical pumps that can fail for an assortment of reasons. Utilizing turnout gear as the diffusion membrane allows direct measurement of volatile chemicals passing through the gear without the need to involve human subjects.

The Joint Service Lightweight Integrated Suit Technology (JSLIST) employs either a chemical protective undergarment (CPU) or vapor protective fire-retardant undergarment (VPFRU) as chemical vapor barriers [14]. The CPU and VPFRU were made from a single layer carbon non-woven fabric. In the study reported here, a woven activated carbon layer (ACL) was incorporated into the passive sampling dosimeter rather than a selective permeable membrane. Multiple compounds from the partial combustion and pyrolysis produced from burning select substrate materials were used to test the adsorption of the compounds onto an ACL. Experiments employing a passive sampling dosimeter and a compound identification application demonstrated that EPA priority pollutant compounds permeate through the turnout gear used in this study; however, most of these compounds may be adsorbed onto an ACL.

## 2. Materials and Methods

### 2.1. Development of Passive Sampling Dosimeter

**Figure 1:**
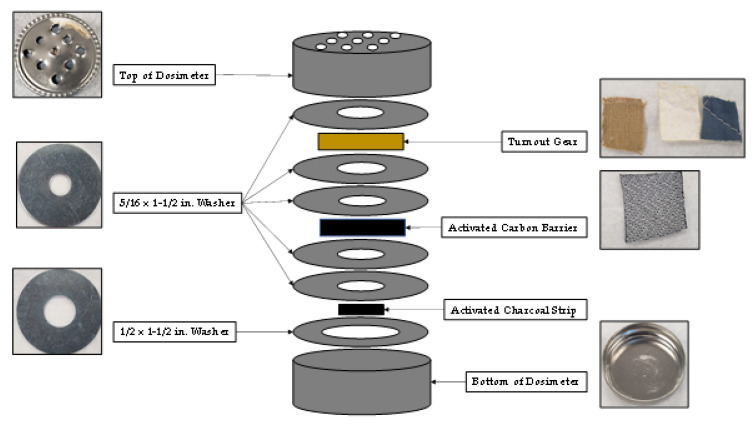
Diagram of passive sampling dosimeter containing turnout gear, ACL, and activated charcoal strip.

The passive sampling dosimeter developed to study compound adsorption and diffusion through turnout gear is shown in Figure 1. The top and bottom of the dosimeter was composed of a 1/2 oz. specimen tin can (Electron Microscopy Sciences, Hatfield, PA, USA). Nine holes were drilled into the top of the dosimeter to allow vapors from the fire to enter the apparatus. Five 5/16 in. × 1- 1/2 in. zinc fender washers and one 1/2 in. × 1- 1/2 in. zinc fender washer (Hillman, Cincinnati, OH) were used in the dosimeter to establish a diffusion path of controlled length. One 5/16 in. × 1- 1/2 in. zinc fender washer was placed between the top of the dosimeter and the retired turnout gear provided by Osceola County Fire Rescue (Kissimmee, FL). The turnout gear contains three-layers: (1) an outer shell composed of 40% Basofil^®^ and 60% Kevlar^®^, (2) a moisture barrier composed of polytetrafluoroethylene (PTFE) and Nomex^®^ needle fabric, and (3) a thermal layer composed of spun Nomex^®^. All three layers of the turnout gear (outer shell, moisture barrier and thermal liner) were simultaneously mounted in the dosimeter during each test. Two more 5/16 in. × 1- 1/2 in. zinc fender washers were placed behind the turnout gear. An optional ACL (Shreddies, Leicestershire, England) composed of activated carbon cloth was then placed behind the two washers, and above two more 5/16 in. × 1- 1/2 in. zinc fender washers. When the optional ACL was not used in the dosimeter four washers were placed behind the turnout gear to keep the diffusion path through the dosimeter consistent. An activated charcoal strip (Albrayco Technologies Inc, Cromwell, CT, USA) was placed in a 1/2 in. × 1- 1/2 in. zinc fender washer at the bottom of the dosimeter, and the apparatus was closed to be used for analysis.

Proper functioning of the dosimeter was tested by placing an impermeable membrane made of a thin piece of aluminum foil (not shown in Figure 1) in the diffusion path behind the turnout gear and before the second washer. The purpose of the aluminum foil was to ensure that vapor phase analytes were not diffusing around the washers to reach the activated charcoal strip.

### 2.2. Burn Method and Extraction

A modified destructive distillation method (MDDM) was utilized to produce the partial combustion and pyrolysis compounds from substrate materials in these experiments. Two substrates, vinyl siding and a railroad tie (rationale for the selection of these substrates is addressed below), were placed in an unlined quart paint can (BestContainers.com, accessed on 20 April 2021, Eagle, ID) and loosely closed with a compression lid containing nine drilled holes in the top of the can. The substrates were positioned at the bottom of the can and heated by applying a propane torch to the bottom external surface of the can. After smoke first appeared from the holes of the lid, the substrates were heated for two additional minutes. Once heating ceased, a lid containing no holes was loosely placed over the can until it cooled to room temperature. Two dosimeters, one containing an ACL and one without an ACL, and a 1 cm × 2 cm activated charcoal strip suspended on a paperclip with unwaxed dental floss were then placed in the can for 15 min. After 15 min, the two dosimeters and suspended activated charcoal strip were removed from the can. Compounds adsorbed on the turnout gear, activated charcoal strips, and ACL that were contained in the can were extracted for analysis. Approximately 0.5 mL of low benzene carbon disulfide (Thermo Fisher Scientific, Waltham, MA, USA) were added to 4 mL glass extraction vials (Thermo Fisher Scientific, Waltham, MA, USA) containing each sample from the dosimeter apparatus and the suspended activated charcoal strip. Extraction was allowed to proceed for 5 min without heating or sonication. The carbon disulfide (CS_2_) was then removed from the extraction vials, and placed in 400 µL vial inserts (Agilent Technologies, Santa Clara, CA, USA). The inserts were placed into 2 mL vials (Thermo Fisher Scientific, Waltham, MA, USA) and sealed with 11 mm crimp seal caps (Thermo Fisher Scientific, Waltham, MA, USA) to be analyzed by gas chromatography–mass spectrometry (GC-MS). The experiments with vinyl siding and a railroad tie were repeated in triplicate.

### 2.3. GC-MS Parameters

All analyses were performed on an Agilent 7890A gas chromatograph with a G45567A series autosampler connected to a 5977E mass spectrometer. Chromatographic separation was performed on a HP-1 methyl siloxane column (25 m length × 0.20 mm internal diameter × 0.5 µm film thickness) with helium carrier gas at a constant flow rate of 0.77 mL/min. The inlet liner used for this experiment was an ultra inert liner, splitless, single taper with no wool (Agilent Technologies, Santa Clara, CA, USA). Sample injections of 1 µL were split 50:1 on injection into a 250 °C injection port. An initial GC oven temperature of 50 °C was held for 3 min and then ramped at 10 °C/min to a final temperature of 280 °C and held for 4 min for a total run time of 30 min. The transfer line to between the GC and MS was held at 280 °C. Mass spectra were collected over a scan range of 30–350 *m*/*z*. The quadrupole temperature of the mass spectrometer was 150 °C and the source temperature was 230 °C.

### 2.4. Keyence Microscopy Parameters

A Keyence VHX-6000 digital microscope with a dual-objective zoom lens VH-ZST was used for microscopic analysis of the ACL. A full ring light shift provided lighting of the materials. A depth composition and 3D display function captured images of the materials composing the ACL.

### 2.5. Compound Identification

The data analyzed for compound identification and frequency of occurrence in this study was from the Ignitable Liquid Reference Collection (ILRC) and Substrate databases [9,10]. The databases were developed by the National Center for Forensic Science (NCFS) and the Technical/Scientific Working Group for Fire and Explosion Analysis (T/SWGFEX). The parameters for sample preparation and GC-MS analysis can be found on the referenced database websites. The datasets contain 1047 ignitable liquids, both weathered and unweathered, and 553 substrates burned for 2 min using the Modified Destructive Distillation Method (MDDM). The sample name, filename, and ASTM subclass (for ignitable liquids only) served as identifiers for each sample. Data organization and compilation was performed in Microsoft Excel (Microsoft Corporation, Seattle, WA) and a novel application was developed in R [15] to use target factor analysis (TFA) for the automated detection and identification of compounds within each sample using target factors comprised of the mass spectra from the NCFS library, as discussed below.

Peaks were detected in the total ion chromatogram (TIC) and extracted ion profiles (EIPs) based on the second derivative with intensities that exceeded ±1 standard deviation from the mean. Derivatives were numerically calculated using the Savitzky–Golay algorithm [16] implemented in the signal package in R [17]. The retention times (RTs) for the chromatogram were converted to retention index (RI) using a cubic spline interpolation of the retention times of the n-alkanes in ASTM E1618 Test Mix standard SRM 2285. The cubic spline interpolation modeled the conversion of RT to RI over the entire range of retention times, including the portions of the range before n-pentane and after eicosane. For a peak to be identified as one of the 295 targeted compounds, the peak had to be within a retention index range of ±30 RI units of the targeted compound and have a Pearson correlation of 0.99 between the target compound mass spectrum and the mass spectrum recovered from projecting the target mass spectrum into the principal component space derived from analysis of spectra comprising the peak. Target factor analysis is discussed below.

The optimal values of: (1) the number of standard deviations from the mean of the second derivative ∈ {1, 2, 3, 4, 5, 6, 7, 8}, (2) the retention index range ∈ {±5, ±10, ±15, ±20, ±25, ±30}, and (3) Pearson correlation for peak identification ∈ {0.701, 0.761, 0.884, 0.989, 0.990, 0.991, 0.992, 0.993, 0.994, 0.995, 0.996, 0.997, 0.998, 0.999, 1.00} were determined using a full factorial analysis of these three factors. The true positive rate (TPR) was calculated for each of the 720 combinations of the three factors. The correct identification of one of the five major peaks identified previously in each sample in the NCFS databases was considered a true positive, and the TPR was calculated by taking the total number of true positives divided by the true positives and false negatives identified in the NCFS database samples. The highest TPR of 0.752 was determined for the set of variables containing a Pearson correlation of 0.99 or greater, ±1 standard deviation from the mean of the chromatographic second derivative, and a retention index range of ±30 RI.

Compound identification was performed by analyzing 11 consecutive mass spectral scans centered on a peak maximum. Identification was accomplished utilizing TFA, a statistical method for determining whether an independent target test vector can be excluded as a contributing factor in collection of data. TFA, as described in previous literature [18,19,20], reduces the dimensionality of a data set and identifies abstract factors. Abstract principal factors required to reproduce the data structure without reproducing the noise are identified and then further transformed to identify individual factors that are physically meaningful (i.e., spectra). The number of principal factors retained for TFA was identified using the determination of rank by median absolute deviation (DRMAD) [21]. DRMAD identifies the error in the dataset by calculating the relative standard deviation of the error eigenvalues.

The following equation shows how the composition of the data matrix [D] is expressed in terms of a linear combination of abstract factors and error:

(1) $$[D]=\left[R^{‡}\right]\left[C^{‡}\right]+\left[E\right]$$

The data matrix [D] contains rows that correspond to each of the 11 consecutive scans across a selected peak, and columns that correspond to nominal *m*/*z* ratios between 30 and 200 from each mass spectral scan. The matrices $\left[R^{‡}\right]$ and $\left[C^{‡}\right]$ contain the scores and loadings for the number of principal factors derived from analysis of [D] and determined by DRMAD. The error matrix, [E], contains the error or noise removed from the data after determining the principal factors of the dataset. A target factor rotation is then used to compare each of the 295 compounds from the NCFS library which fall within ±30 RI units of the peak maximum. This process is repeated for each peak in the chromatogram. The vector transformation is described in Equations (2)–(4) below.

(2) $$\left[D\right]=\left[R^{‡}\right]\left[T\right]{\left[T\right]}^{-1}\left[C^{‡}\right]$$

(3) $$T_{l}^{’}=\overset{=}{C_{l}}\;{\left[C^{‡}\right]}^{T}$$

(4) $$\bar{C_{l}}{=T}_{l}^{’}\left[C^{‡}\right]$$

In Equation (2), [T] is the transformation matrix used to produce a meaningful oblique rotation of $\left[C^{‡}\right]$ and the error contribution, [E], has been dropped. It is generally not possible and not required to find the entire transformation matrix, [T]. Instead, it suffices to find acceptable test factors (vectors) one at a time. In Equation (3), $T_{l}^{’}$ is a transformation vector for the lth row of ${\left[T\right]}^{-1}$, and $\overset{=}{C_{l}}$ is the spectrum vector of one of the 295 target compounds in the NCFS library which meets the RI proximity requirement. In Equation (3), ${\left[C^{‡}\right]}^{T}$ is the inverse of $\left[C^{‡}\right]$ which is composed of an orthonormal set of vectors. Equation (4) then calculates $\bar{C_{l}}$ from $\overset{=}{C_{l}}$ and a comparison of the two vectors determines if the vector $\overset{=}{C_{l}}$ projected into the abstract factor space lies within the space defined by the principal factors. The comparison of $\bar{C_{l}}$ and $\overset{=}{C_{l}}$ was made by calculating the Pearson product moment correlation. A Pearson correlation of 0.99 or greater was required to identify a compound corresponding to $\overset{=}{C_{l}}$ as contributing to the chromatographic peak.

## 3. Results

### 3.1. Microscopic Analysis of ACL

**Figure 2:**
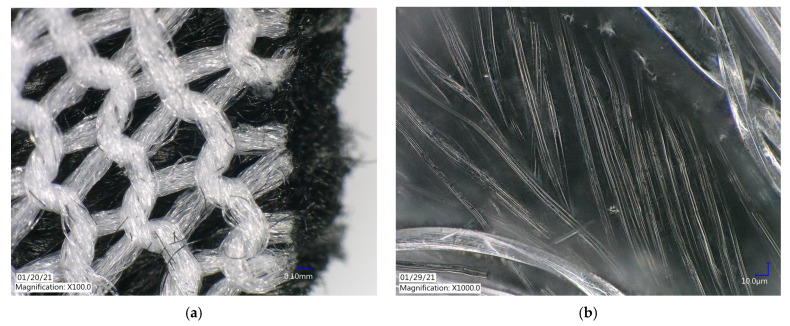
ACL under Keyence VHX-6000 digital microscope at (**a**) ×100 magnification and (**b**) ×1000 magnification.

The ACL was imaged using the Keyence VHX-6000 digital microscope to visualize the physical structure of the material. The activated carbon material, previously reported to be a highly porous material [22], was formed into threads approximately 5 µm in width. The threads were then woven into a panel to create the cloth barrier used in the apparatus (Figure 2).

### 3.2. Carcinogenic Compound Identification and Frequencies

#### 3.2.1. Frequency of Priority Pollutants

The 295 compounds in the NCFS library were compared to the Priority Pollutant List [4] released by the United States Environmental Protection Agency (EPA). There were 14 compounds common between the two lists that are listed in Table 1 along with the occurrence frequencies of each of the compounds in the 1047 ignitable liquid and 553 substrate samples. The frequencies listed in Table 1 incorporate every occurrence of the compound identified in a peak of sufficient intensity using TFA if the Pearson correlation had a value above 0.99 and is ±30 RI units of the targeted compound. This method can identify a peak as more than one compound if the criteria for each compound identification is met. The frequencies in Table 1. Frequencies of priority pollutant compounds identified in 1047 ignitable liquids and 553 substrates found in ILRC and Substrate databases should be regarded as upper limits.

The 14 compounds in Table 1 were used as a guideline to choose two substrates for the MDDM burns which would produce multiple compounds from the table. Heating of vinyl siding and a railroad tie were used because the vinyl siding produces benzene, toluene, ethylbenzene, and naphthalene from the table above, while the railroad tie produces naphthalene, 2-methylnaphthalene, acenaphthene, and fluorene. Partial combustion and pyrolysis products from the two substrates cover a retention index range of 660–1575 which proved useful in examining the diffusion of a range of compounds with different molecular weights through the turnout gear.

#### 3.2.2. Compound Identification

Compound identification was focused on seven major compounds from Table 1 in the previous section: benzene, toluene, ethylbenzene, naphthalene, 2-methylnaphthalene, acenaphthene, and fluorene. The following figures are normalized to the most intense peak of the TIC.

**Figure 3:**
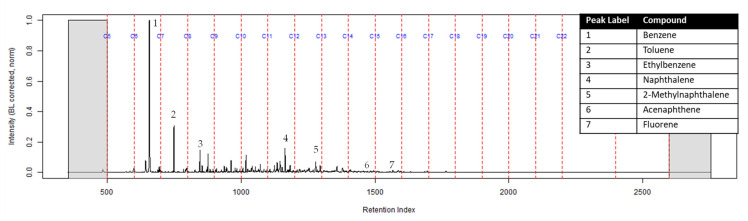
TIC of the CS_2_ extract from the suspended activated charcoal strip from can containing 2-min MDDM burned vinyl siding and a railroad tie (maximum peak abundance 1.8 × 10^7^).

**Figure 4:**
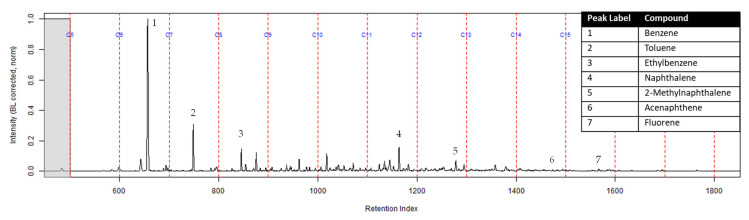
Zoomed TIC of the extract from the suspended activated charcoal strip from a can containing 2-min MDDM burned vinyl siding and a railroad tie (maximum peak abundance 1.8 × 10^7^). This charcoal strip was not in a dosimeter.

The TIC of the CS_2_ extract from the activated charcoal strip suspended in the headspace of the can with the identification of the seven compounds is shown in Figure 3 (full range) and Figure 4 (area of interest).

**Figure 5:**
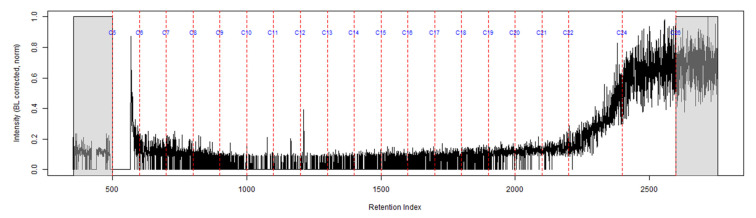
TIC of the extract from the turnout gear within the dosimeter from the can containing 2-min MDDM burned vinyl siding and a railroad tie (maximum peak abundance 2.5 × 10^5^).

The TIC from the GC-MS analysis of the extract of the turnout gear placed in the dosimeter without the ACL is shown in Figure 5. No compounds were detected from the turnout gear which is demonstrated from the background noise and column bleed. This indicates that no chemical vapors were adsorbed onto the turnout gear. Similar results were obtained for the turnout gear placed in the dosimeter with the ACL.

**Figure 6:**
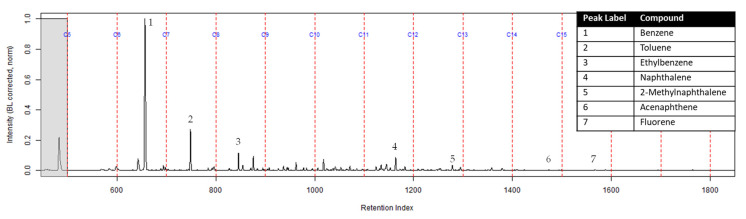
Zoomed TIC of the extract from the activated charcoal strip within the dosimeter not containing ACL from the can containing 2-min MDDM burned vinyl siding and a railroad tie (maximum peak abundance 5.2 × 10^6^).

The TIC of the extract from the activated charcoal strip in the dosimeter that did not contain the ACL is shown in Figure 6. All seven compounds of interest were identified indicating they diffused through the turnout gear and were adsorbed by the activated charcoal strip.

**Figure 7:**
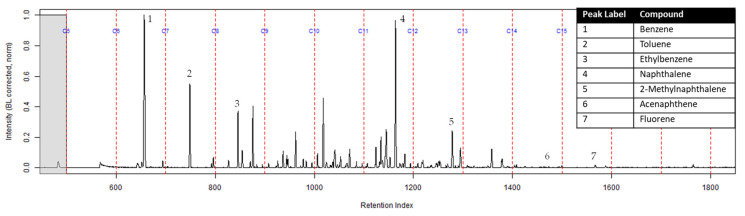
Zoomed TIC of the extract from the ACL within the dosimeter from the can containing 2-min MDDM burned vinyl siding and a railroad tie (maximum peak abundance 4.8 × 10^6^).

**Figure 8:**
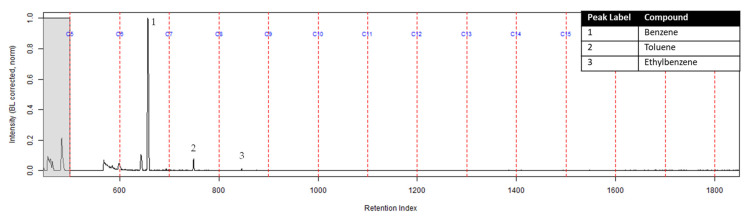
Zoomed TIC of the CS_2_ extract from the activated charcoal strip within the dosimeter containing the ACL from the can containing 2-min MDDM burned vinyl siding and a railroad tie (maximum peak abundance 1.2 × 10^6^).

The TICs of the extracts from the ACL and the activated charcoal strip from the dosimeter containing the ACL are shown in Figure 7 and Figure 8 respectively. All seven compounds of interest were identified on the ACL using TFA. However, the lower molecular weight compounds had a lower peak intensity in comparison to the higher molecular weight compounds relative to the ratio that was calculated on the extract from the suspended activated charcoal strip from Figure 4. Figure 8 shows the TIC for the extract from the activated charcoal strip behind the ACL in the dosimeter. Three of the lighter molecular weight compounds were identified on the activated charcoal strip. This result demonstrates that the lighter molecular weight compounds diffused through the turnout gear and the ACL before being sampled on the activated charcoal strip.

When the experiments were repeated with an aluminum foil barrier behind the turnout gear, analytes were not detected on either the ACL or activated carbon strip. This result demonstrates proper performance of the dosimeter as an air-tight sampling device that was not “leaking” analytes around the washers, see Figure 1.

Extraction of similar chemical components from the turnout gear was tested by addition of a 10 μL volume of a medium (C10–C15) naphthenic paraffinic ignitable liquid (SRN 835 from the Ignitable Liquids Reference Collection and Database, see reference 9) was added to a swatch of the turnout gear (all three layers) and allowed to stand for five minutes in the face of a chemical fume hood prior to extraction. The turnout gear was extracted following the same protocol used in the dosimeter experiments and the GC-MS TIC was compared with that of the neat liquid. All components of the ignitable liquid sample were recovered from the turnout gear.

## 4. Discussion

### 4.1. Frequency of Priority Pollutants and Fire Debris Compounds

The occurrence frequencies of the 14 compounds in Table 1 were calculated based on the TFA analysis of 1047 ignitable liquids and 553 substrates from the ILRC and Substrate databases. Seven of the compounds were identified in 10% or more of the ignitable liquids or substrates in the databases. Examples of substrates from the Substrate database containing these compounds can be found in Table 2 below.

### 4.2. Compound Identification

The extract of the activated charcoal strip suspended in the can containing the heated vinyl siding and railroad tie had the seven compounds of interest for this study (Table 1) identified using TFA. None of the seven compounds of interest were identified from the extracts of the turnout gear. All seven compounds of interest were identified in the extracts from the ACL (where applicable) and the activated charcoal strip within the dosimeter not containing the ACL. Three of the compounds, benzene, toluene, and ethylbenzene, were identified in the extract from the activated charcoal strip behind the ACL.

The dosimeter without an ACL was used to determine whether chemical vapors were being adsorbed onto the turnout gear or passing through to the firefighter. The extract of the turnout gear from the dosimeter without the ACL did not contain any adsorbed compounds from the heated substrates; however, the extract of the activated charcoal strip within the dosimeter did contain all seven compounds of interest. Therefore, the turnout gear in this study does not prevent carcinogenic chemical vapors from diffusing through the fabric to a firefighter’s skin. This demonstrates that the retired turnout gear used in this study, manufactured under NFPA 1971 [1], allows for diffusion of chemical vapors. Additional studies need to be performed with new and used turnout gear from multiple sources to confirm the generality of these results.

The dosimeter with an ACL was used to determine whether an ACL would adsorb the chemical vapors and provide the firefighter with some protection. No compounds were detected from the extract of the turnout gear demonstrating again that none of the carcinogenic compounds adsorbed onto the turnout gear. All seven compounds of interest adsorbed onto the ACL placed behind the turnout gear; however, the relative abundance between the compounds differed from the abundances observed from the suspended activated charcoal strip in the can. Benzene, toluene, and ethylbenzene were identified in the extract of the activated charcoal strip located behind the ACL. These results indicate that the lower molecular weight compounds partially diffused through the ACL and were adsorbed onto the activated charcoal strip behind the ACL. In the TIC for the CS_2_ extract of the suspended activated charcoal strip (Figure 4) the peak ratio for benzene:toluene is 3:1 and the ratio for benzene:naphthalene is 5:1. The same peak ratios are present in the TIC for the extract of the activated charcoal strip in the dosimeter without an ACL (Figure 6). In Figure 7 the TIC for the extract of the ACL has a peak intensity ratio for benzene:toluene of 2:1 and benzene:naphthalene of 1:1. Figure 8 shows the TIC for the extract of the activated charcoal strip in the dosimeter with the ACL and has a peak intensity ratio for benzene:toluene of 10:1 while naphthalene is not present in the chromatogram. The peak ratios obtained for Figure 7 and Figure 8 further lead to the conclusion that heavier compounds are being adsorbed onto the ACL completely, while a portion of the lighter compounds are diffusing through, with benzene diffusing through the most. This could potentially be due to the overloading effects of activated carbon which has been studied previously [23]. In previous studies it was found that when activated carbon is being overloaded with compounds, the lighter molecular weight compounds are displaced by heavier molecular weight compounds. Compound displacement on the ACL could explain a portion of the lighter molecular weight compounds having diffused through the barrier to the activated charcoal strip. This is prominent when noting the differences in compound intensities compared to one another in each chromatogram. Therefore, the ACL has the potential to be used with turnout gear to prevent or decrease the quantities of carcinogenic compounds diffusing through the turnout gear and onto the skin of firefighters. Activated carbon, as a fabric, should be studied further to determine the strengths and limitations of incorporating the fabric with turnout gear.

## 5. Conclusions

Turnout gear used as a passive sampling diffusion ‘membrane’ in this study allowed for diffusion of chemical vapors, including carcinogens, present at a fire scene. It is important to identify and study potential approaches to protect the firefighter from carcinogenic compounds that may diffuse through turnout gear. Results from this study also demonstrate potential benefits from an activated carbon layer.

## Figures and Tables

**Table 1:** Frequencies of priority pollutant compounds identified in 1047 ignitable liquids and 553 substrates found in ILRC and Substrate databases.

Compound	Frequency in Ignitable Liquids (%)	Frequency in Substrates (%)
Acrolein	1	1
Methylene chloride	1	1
1,2-Dichloroethane	0	1
Benzene ^1^	1	18
Toluene ^1^	30	83
Ethylbenzene ^1^	25	59
Bis(2-chloroethyl) ether	0	1
Phenol	1	23
Naphthalene ^1^	24	48
Acenaphthene ^1^	1	1
Diethyl phthalate	4	1
Fluorene ^1^	7	31
Anthracene	22	6
Dibutyl phthalate	0	3

^1^ Compounds identified in substrates used in this study.

**Table 2:** Examples of substrates from the Substrate data containing priority pollutant compounds that were contained in 10% or more of the ILRC and/or Substrate Database samples.

Compound	Examples of Substrates
Benzene	Cotton towel, vinyl siding, polystyrene ceiling tiles, automobile car seats, roofing paper
Toluene	Polyester carpet, thermal paper, vinyl siding, polystyrene ceiling tiles, window blinds
Ethylbenzene	Magazines, gel pens, vinyl siding, automobile tires, window blinds
Phenol	Polyurethane mattress pads, bamboo hardwood, cotton paper, laminate flooring
Naphthalene	Nylon carpet, cork tiles, yellow pine wood, vinyl siding, railroad tie, plastic clothesline
Fluorene	Polyester carpet, railroad tie, polyester quilt batting, alder wood
Anthracene	Polyester carpet, railroad tie

## Data Availability

The data presented in this study are available in the NCFS databases [9,10].
